# Supplementary material for: Quantitative comparison of PI(3,5)P2 biosensors reveals SnxA is the most sensitive and unbiased
Source: Mol Biol Cell. 2026 Sep 14;37(10):ar91. doi: 10.1091/mbc.E26-03-0120 (PMC13637729; doi:10.1091/mbc.E26-03-0120)
Supplement: Supplementary file 1 [file reviewer_reportE26-03-0120.pdf]

Quantitative comparison of PI(3,5)P2 biosensors reveals SnxA is the most sensitive and unbiased

Gerald Hammond, Claire Weckerly, and Tiernan Swayhoover

Corresponding author(s): Gerald Hammond, University of Pittsburgh School of Medicine

Review Timeline:

Submission Date:	2026-03-09
Editorial Decision:	2026-03-16
Revision Received:	2026-07-10
Accepted:	2026-07-28

Editor-in-Chief: John Aitchison

Transaction Report:

(Note: With the exception of the correction of typographical or spelling errors that could be a source of ambiguity, letters and reports are not edited. The original formatting of letters and referee reports may not be reflected in this compilation.)

RE: Manuscript #E26-03-0120
TITLE: Quantitative comparison of PI(3,5)P2 biosensors reveals SnxA is the most sensitive and unbiased

Dear Dr. Hammond:

Thank you for considering Molecular Biology of the Cell for your revised Review Commons manuscript. The revision plan you have outlined is logical, and we would be happy to consider a revised version of the manuscript that follows that plan.

Sincerely,

Laura Lackner
Monitoring Editor
Molecular Biology of the Cell

Dear Dr. Hammond,

The review of your manuscript, referenced above, is now complete. The Monitoring Editor has decided that your manuscript is not acceptable for publication at this time, but may be deemed acceptable after specific revisions are made, as described in the Monitoring Editor's decision letter above and the reviewer comments below.

A reminder: Please do not contact the Monitoring Editor directly regarding your manuscript. If you have any questions regarding the review process or the decision, please contact the MBoC Editorial Office (mboc@ascb.org).

When submitting your revision include a rebuttal letter that details, point-by-point, how the Monitoring Editor's and reviewers' comments have been addressed. (The file type for this letter must be "rebuttal letter"; do not include your response to the Monitoring Editor and reviewers in a "cover letter.") Please bear in mind that your rebuttal letter will be published with your paper if it is accepted, unless you have opted out of publishing the review history.

Authors are allowed 90 days to submit a revision. If this time period is inadequate, please contact us at mboc@ascb.org.

Revised manuscripts are assigned to the original Monitoring Editor whenever possible. However, special circumstances may preclude this. Also, revised manuscripts are often sent out for re-review, usually to the original reviewers when possible. The Monitoring Editor may solicit additional reviews if it is deemed necessary to render a completely informed decision.

In preparing your revised manuscript, please follow the instruction in the Information for Authors (www.molbiolcell.org/info-for-authors). In particular, to prepare for the possible acceptance of your revised manuscript, submit final, publication-quality figures with your revision as described.

To submit the rebuttal letter, revised manuscript, and figures, use this link: Link Not Available

MBoC offers the option to publish your work with immediate open access. Open access can increase the discoverability and usability of your research. If you would like to publish your paper with immediate open access but did not select this option during initial submission, please contact the editorial office at mbc@ascb.org.

Authors of Articles and Brief Communications are also encouraged to create a short video abstract to accompany their article when it is published. These video abstracts, known as Science Sketches, are up to 2 minutes long and will be published on YouTube and then embedded in the article abstract. Science Sketch Editors on the MBoC Editorial Board will guide you as you prepare your video. Information about how to prepare and submit a video abstract is available at www.molbiolcell.org/science-sketches. Please contact mboc@ascb.org if you are interested in creating a Science Sketch.

Sign up for MBoC's new content alerts here https://tinyurl.com/3bcsyk3e

Thank you for submitting your manuscript to MBoC. We look forward to receiving your revised paper.

Sincerely,

Eric Baker
Journal Production Manager
MBoC Editorial Office

mbc@ascb.org

Response to Reviewers

Manuscript number: RC-2026-03394
Corresponding author(s): Hammond, Gerald

1. General Statements [optional]

As you can see from the reviewer's comments, there is substantial enthusiasm for our manuscript, which adds "gold standard" evidence for the veracity of the new $PI(3,5)P_2$ probe, SnxA. $PI(3,5)P_2$ is a low abundance lipid with nevertheless fundamental roles in cell biology and implications in the progression – and treatment – of neurological disease. Therefore, we believe the work warrants publication in a suitable broad-readership cell biology journal like *Molecular Biology of the Cell*. Indeed, the reviewers found our work to be *"a strong study with vigorous and well controlled experiments. It is of broad interest to the cell biology community"* (reviewer 1), "*done very thoroughly, and considering the increasing interest in the less studied phosphoinositol species, it is also of significant interest"* (reviewer 2) and *"represents a compelling and well-executed cell biological analysis that … <u>will facilitate future investigations into the still poorly understood mechanisms regulating $PI(3,5)P_2$ metabolism</u>"* (reviewer 3). We hope that you as editor agree, and will consider publication of the manuscript after completion of the proposed revisions detailed herein.

We are grateful to all three reviewers, who identified areas where the manuscript could be further strengthened. We have paid heed to all of their insights, as detailed below:

2. Description of the revisions

Reviewer 1

#1.1 In Fig 1 it appears that both PI3P and PI3,5P2 are present on lysosomes (Lamp1-FRB) and that recruitment of MTM1 (via FKBP) depletes both of them. Additionally, In the images and quantifications, it appears that after recruitment of MTM1, there is no vesicular pools of PI3P or PI3,5P2 left at all. This raises several concerns:

#1.1a What is happening to the pool of PI3P on Rab5 endosomes?

We have now added new data to figure 1, showing robust depletion of FYVE-EEA1 (and SnxA) by recruitment to FRB-Rab5 or LAMP1, as described in the **first paragraph of p. 3**.

#1.1b The main pool of PI3P is on Rab5 endosomes and separate from LAMP1 lysosomes. Why does recruitment of MTM1 to lysosomes then lead to almost complete depletion of all PI3P (Fig1 B bottom panel), when the lysosomal pool of this lipid should be minor compared to that on early endosomes?

We have answered this question in the results section on the **last paragraph on p 2**, explicitly in the following: *"FYVE-EEA1 was completely eliminated from all visible membranes by FKBP-MTM1 recruitment to either FRB-RAB5 or LAMP1-FRB (**Fig. 1B**), despite being much more enriched at the Rab5-positive membranes (**Fig. 1C**)."* and later in the same paragraph: *"the loss of predominantly Rab5-localized FYVE by recruitment of MTM1 to LAMP1 was unexpected. Given the depletion of the probe occurred in less than the 30 s image interval (**Fig. 1B**), we*

speculate that a low level of LAMP1-FRB present in sorting endosomes may have recruited enough FKBP-MTM1 to deplete PI3P."

#1.1c The main problem comes from disagreement with figure 3. There it is shown that ".PI(3,5)P2 does not significantly overlap with PI3P". If this is true, then why does recruitment of MTM1 to LAMP1 lead to the depletion of both PI3P and PI3,5P2. This would indicate that they are on the same compartment (LAMP1 positive lysosomes)?

This question is explicitly addressed in the results description for the new figure 1 in the **last paragraph on p 2**, particularly:

"These observations preclude conclusions on the localization of the lipid biosensors based on the extent of depletion following enzyme recruitment to either Rab5 or LAMP1. Indeed, we note measurable overlap of both biosensors with only a subset of Rab5 or LAMP1-positive membranes – with the exception of FYVE, which was present on all Rab5-positive membranes."

As well as in the **first paragraph of the "PI(3,5)P2 does not significantly overlap with PI3P"** results section on **p. 3**: "Although we saw some degree of overlap of both biosensors with Rab5 and LAMP1 (**Fig. 1C**), we found SnxA only localized to a subset of each, and FYVE only localized to a subset of LAMP1 vesicles (insets in **Fig. 1D**), suggesting they do not reside on the same vesicle."

#1.1d A merge and colocalization analysis is at least needed to address how much of SnxA and EEA1-FYVE is localized to LAMP1-FRB. But just by looking at the one cell depicted, the localisation of LAMP1-FRB seems to be rather distinct from EEA1-FYVE and SnxA

We have quantitative co-localization data added in **figure 1C** for both PI3P and $PI(3,5)P_2$ biosensors.

#1.2 Minor: Fig2 is all in all way to small and it is difficult to actually see the signal in panel A and C. It should also be noted that in these panels (zoomed in to my best ability) that there is clear localisation of SnxA to the plasma membrane. Is this pool lost after Apilimod treatment, or is this unspecific binding? This PM localisation also appears in Fig1 B and 5C and should be discussed.

We have repeated the experiments in figure 2 in response to #1.5. We believe the new images provided in the updated figure, including merged images, make the signal easier to discern. We have also specifically addressed the plasma membrane localization of the SnxA probe at the **end of the section "*$PI(3,5)P_2$ loss precedes vacuolation during apilimod treatment*" on p3** as follows: "Note that SnxA also partially labeled the plasma membrane in some cells (see for example Figs. 1B and 2B, C). However, this weak staining is not depleted by inhibitors, and likely does not reflect a $PI(3,5)P_2$-driven interaction, as noted in the initial description of the sensor (Vines et al., 2023)."

#1.3 It should also be pointed out that dextran will be taken up through macropinocytosis and might follow different endocytic trafficking to conventional receptor.

We have amended the description of the first paragraph of the "*PI(3,5)P2 loss precedes vacuolation during apilimod treatment*" section on **p. 2** with the following: "We stained the

endosomal pathway for one hour with fluorescent dextran, which is passively loaded through fluid phase endocytosis and macropinocytosis."

#1.4 It also seems that the dextran signal decreases after Ap treatment. Is this the case? Has this been quantified? Would that point towards the whole endocytic structure being degraded and not just PI3,5P2?

We believe this reduction is due to simple photobleaching of the dye under time-lapse imaging. The photobleaching is less evident in the new images provided in the new figure 2, since less excitation was used.

#1.5 OPTIONAL: The timeline of PI3,5P2 degradation and vacuolation is a great experiment and very important. However, PI3,5P2 production is critically dependent on prior generation of PI3P. It could be of interest to readers what happens to SnxA localisation when cells are treated with VPS34-IN-1 or Wortmannin. How does loss PI3,5P2 compare between the timing of PIKfyve inhibition and inhibition of PI3K that generate its substrate. The timescales of this might tell us something interesting about the timings of phosphoinositide conversions. Also, treatment with PI3K inhibitors is known to not lead to vacuolisation even though it does also lead to a loss of PI3,5P2, further indicating that there might be more to the vacuolation phenotype than just the loss of PI3,5P2. These experiments would add novelty and detail to the study but do not change the overall findings or conclusions.

We agree with the reviewer that this is a fascinating and important question, which motivated us to repeat the experiments with apilimod to include a new condition using a Vps34 inhibitor. These results are now detailed in the **second paragraph of "*PI(3,5)P$_2$ loss precedes vacuolation during apilimod treatment*" on p. 2**: "inhibiting PI3P synthesis with VPS34-IN-1 caused a similar rapid decrease in SnxA labelled structures **(Fig. 2C)**. When the loss of SnxA was quantified, fitting a one phase decay model determined that SnxA came off of the membranes with a time constant (τ) of approximately 5 minutes with either Vps34 or PIKfyve inhibitor, suggesting continuous, rapid turnover of PI(3,5)P$_2$ and its precursor **(Fig. 2D)**."

#1.6a: Major: Fig3: The distinct localisation of PI3P and PI3,5P2 looks convincing but does not seem to be in line with data in Fig1.

We believe our response to **#1.1c** addresses this point.

#1.6b It should be clearly noted and discussed that the structure in Fig3 B is a nascent macropinosomes (the size and proximity to the PM make this clear) and this transition on macropinosomes as been well characterised in Vines et al 2023

We agree with the reviewer that this structure could easily be a macropinosome, though without a marker, we cannot definitively make this claim. Of course, the same logic applies to declaration that this is an endosome (though HEK293A cells do possess some similarly very large, Rab5+ve sorting endosomes)! <u>We therefore classified this as "EEA1-FYVE positive vesicle"</u>.

#1.7 Major minor: Figure 5 is a very powerful approach to produce a completely independent pool of PI3,5P2. As control, the PIKfyve-CCR-Kina construct should be recruited to mitochondria in the absence of the MavQ construct. This should lead to the generation of PI5P (the other known

product of PIKfyve) without generating PI3,5P2. This would be a useful control to test that SnxA does not bind to PI5P. (As a side note, this method would be great to use to try and find a bone fide PI5P effector. Maybe by isolating mitos and sending them to Mass Spec after altering their PIP composition? But this is obviously a completely different story)

This is an excellent suggestion. We have now added these controls as a new figure panel, **Fig. 5D**. This is also discussed in the results in the **second paragraph of the section "*Production of PI(3,5)P2 at orthogonal membranes is sufficient to recruit SnxA*" on p.4**: "*The resulting production of PI(3,5)P2 then caused robust localization of SnxA at the mitochondria (Fig. 5B), which was not seen when the FKBP-PIKfyve-CCR-Kina-Dead was used (Fig. 5C) or when FKBP-MavQ was omitted (**Fig. 5D**). Therefore, the recruitment of SnxA to the mitochondrial outer membrane depended on both enzymes that convert PI to PI3P and PI3P to PI(3,5)P_2, eliminating the possibility that SnxA was instead binding PI5P rather than PI(3,5)P_2 in this context (though it should be stated, there is no positive control that PIKfyve was able to generate PI5P from PI at the mitochondrial outer membrane).*"

#1.8 Major: Figure 6 is great work, but it must be discussed that SnxA is a constitutive dimer while the 2xPx is a monomer. Therefore, the SnxA dimer will also contain two fluorescent proteins and will therefore also appear brighter than the 2xPx. Therefore, the increased baseline of SnxA might just be due to its nature as dimer and not because it has a higher affinity to PI3,5P2 compared to the 2xPx. OPTIONAL: One way to clear this up would be to make a 2xPx construct that also contains 2 fluorescent proteins. Also, the increase in fluorescence should also be shown as normalized to the initial intensity. From the graphs in 6a it seems that both have an ~1.3 and ~1.5 fold increase?

This is an important nuance omitted from the original manuscript. We now add the following discussion paragraph on **p. 5**: "*The difference between full-length SnxA and 2xPX-SnxA is surprising, since the difference in affinity for PI(3,5)P_2 in artificial membranes is very small (Vines et al., 2023). Indeed, the full-length SnxA includes a coiled-coil domain that likely induces dimerization, so both probes are in effect dimeric PI(3,5)P_2-binding PX domains. One trivial explanation could be that the dimer of SnxA proteins includes two copies of the fluorescent protein tag, effectively increasing signal to noise. However, this is unlikely to account for the rather large difference in both basal enrichment and PIKfyve-induced endosome recruitment observed in **Fig. 6**. Instead, we speculate that the coiled-coil orientates the two PX domains in a more favorable relative conformation for concurrent PI(3,5)P_2 binding compared to 2xPX-SnxA. In this context, it will be interesting to observe the effects of the recently identified higher-affinity G13V mutant in both probe configurations (Nishimura et al., 2025).*"

Note, we did not normalize the fluorescence intensity to the initial intensity, since the value reported is already a ratio of the Rab5-associated fluorescence to the rest of the cell; therefore, the values as reported give an indication of the relative enrichment of the probe, showing the SnxA has both a higher baseline and stimulated Rab5 association.

#1.9 OPTIONAL (but important): It would be great to compare the recently described G13V mutant of SnxA that is reported to have a higher affinity to PI3,5P2 (Preprint, Nishimura et al 2025). These experiments would add novelty and detail to the study but do not change the overall findings or conclusions. But as the study sets out to find the best PI3,5P2 probe, this might be very important

We agree that this would be an interesting study, but feel it is outside the scope of the current manuscript. However, we do now cite this important study in the overall discussion of relative affinities in response to the last comment **#1.8**.

#1.10 Minor: In the discussion it is stated that "Vines et al. showed SnxA to have higher affinity for PI(3,5)P2 in vitro compared to 2xPx-SnxA" The Kd (to model membranes with 5% PI3,5P2 in POPC background!) for SnxA there is 187+-13nM and for 2xPx 217+-7 nM. Taking the top and bottom ranges of the uncertainty intervals to 200nM and 210nM respectively. While this is still a slightly higher Kd for SnxA, I am unsure how physiologically relevant this difference is.

This is absolutely true and thank you for noticing this oversight in our interpretation! In the response to **#1.8** we also stated how the modest affinity difference likely does not account for the difference.

#1.11 Minor: In the discussion it is stated that "But, we could observe a short temporal overlap between the two lipids within a single endosome, lasting only a few minutes". If this statement refers to Figure 3B, then it must be clearly stated that this a macropinosome and not be conflated with canonical endosomes

We've now modified the wording here (**p. 6**) to reflect that we cannot distinguish macropinosomes and endosomes in these experiments: "*Consistent with prior work (Vines et al., 2023; Pemberton et al., 2025), we observed little overlap between the two lipids in static images of a population of labelled membranes* ***(Fig. 3)****. But, we could observe a short temporal overlap between the two lipids within a single membrane compartment, lasting only a few minutes.*"

Reviewer 2

#2.1 While the data are by enlarge backed up by the presented data, there are two instances in which controls do not show the desired properties in some figures (but do in others). Specifically, the kinase dead control construct experiments in Figures 4D and 5D suffer from a lack of localization of the FKBP-PIKfyve-CCR-Kinase-Dead construct to the Rab5 endosomes or mitochondria respectively. Without robust localization of the control kinase, the dependency on PI(3,5)P2 synthesis (as opposed to physical kinase mediated recruitment) cannot be excluded. The construct shows robust relocalization in Figure 6 when the 2xPx-SnxA is being evaluated. With the combined evidence shown in the paper, this does not create significant doubt about the overall conclusions, but this issue needs to be addressed.

Thank you for drawing attention to this apparent incongruity. Firstly, we have now amended **Fig. 4D** and **Fig. 6C** to show examples with more clear-cut, unambiguous recruitment of the kinase inactive FKBP-PIKfyve mutant, with no induction of SnxA recruitment:

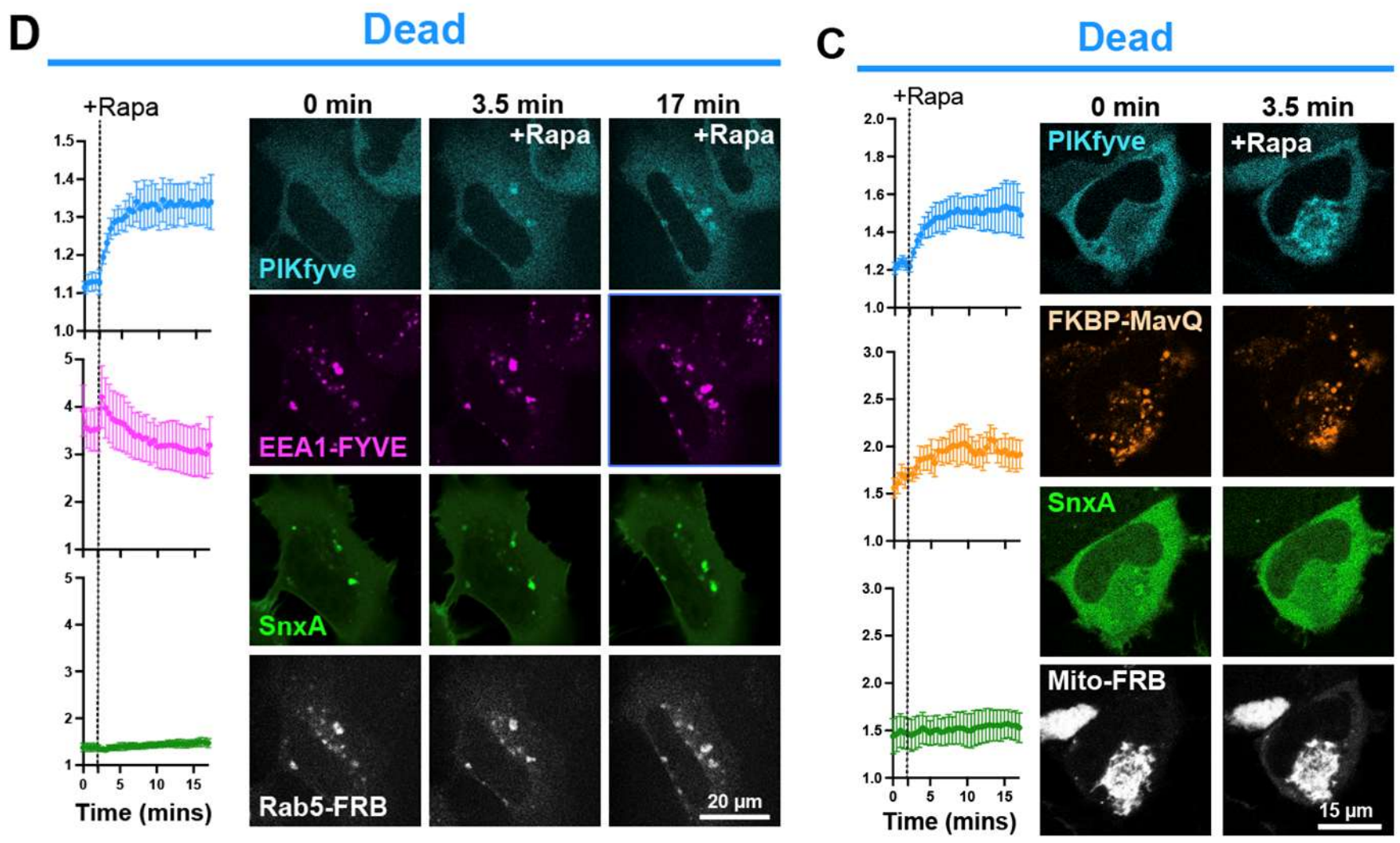

However, these specific examples aside, the quantification indeed shows less average FKBP-PIKfyve^{KD} recruitment than FKBP-PIKfyve^{WT}; therefore, to confirm the difference in SnxA recruitment is indeed due to the lack of catalytic activity, as opposed to simply recruiting less protein, we filtered the data for the active enzyme to analyze solely FKBP-PIKfyve recruitment that was equivalent to the kinase-inactive control, and verified that SnxA recruitment was still observed for the wild type. We include the data here for Rab5 recruitment:

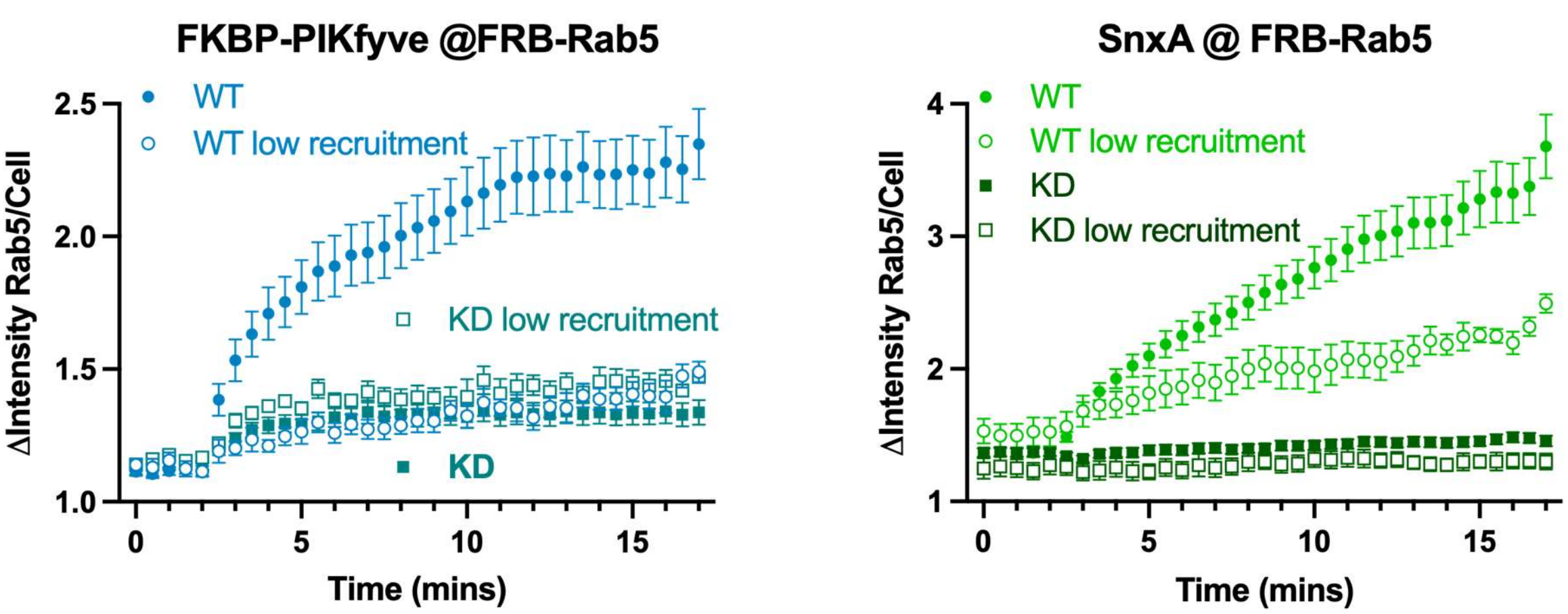

And also, for FRB-Fis1^{tail} mitochondrial recruitment:

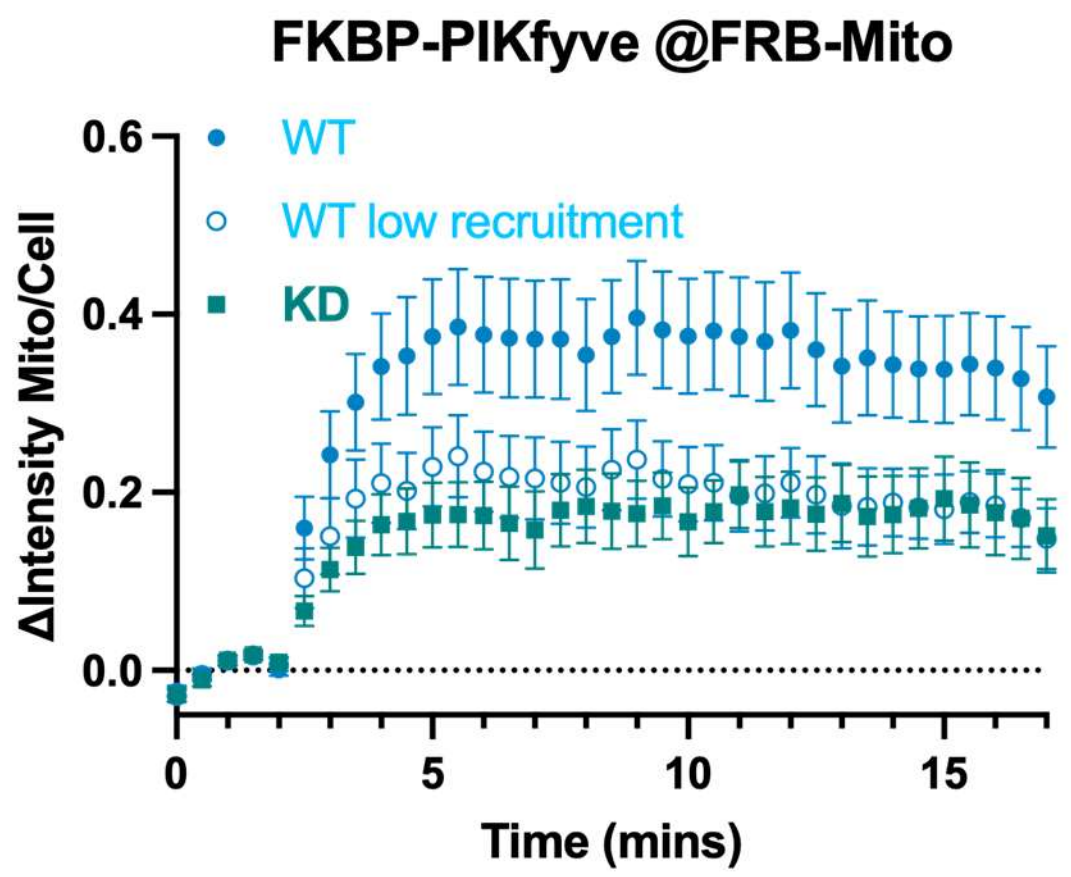

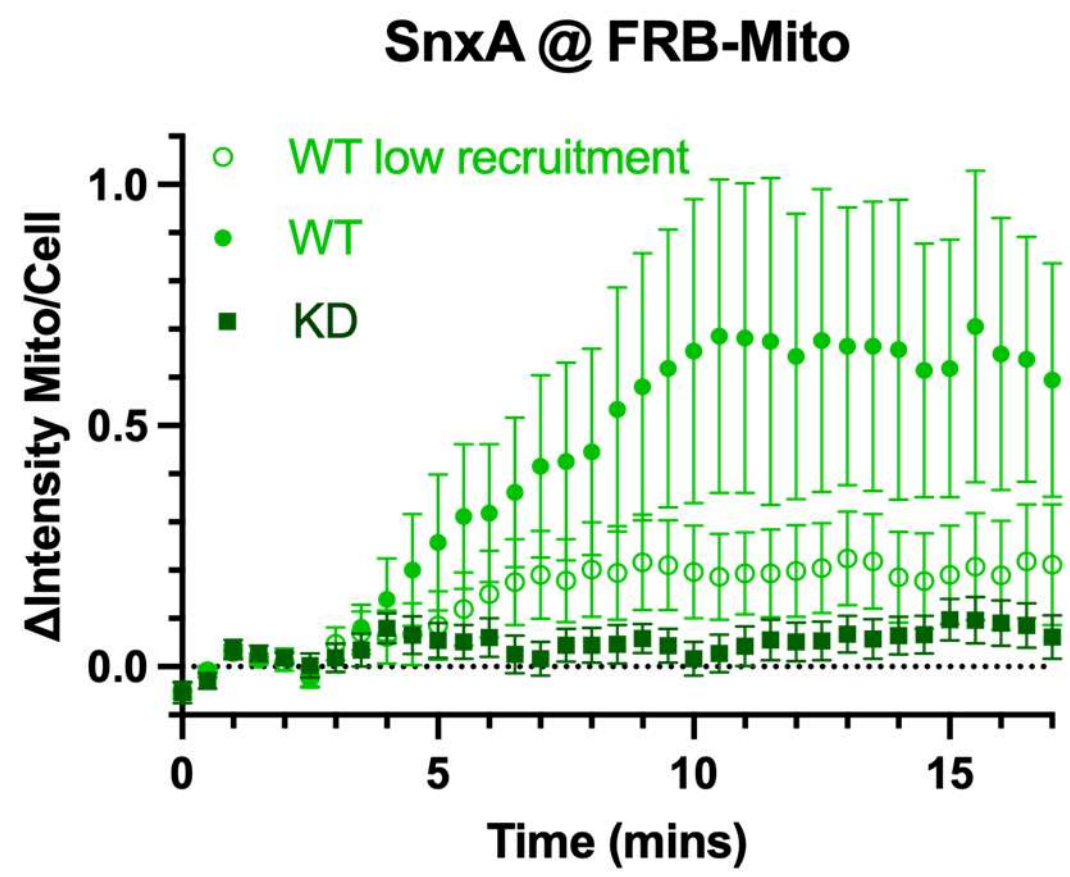

As can be seen, although SnxA recruitment is reduced under these conditions, there is still a level of recruitment not observed in the kinase inactive control. However, in the manuscript we have kept the full data set, since this includes many more wild-type cells than in this filtered analysis.

Reviewer 3

#3.1a In Figure 1B, the authors show a rapid depletion of PI3P upon recruitment of MTM1 to LAMP1-positive membranes. The apparent minimal overlap between EEA1-FYVE and LAMP1 signals in the images is intriguing. It would be helpful if the authors could comment on whether this reflects rapid membrane trafficking dynamics or nonspecific membrane targeting of MTM1.

This is an important point also raised by reviewer 1; our response to ***#1.1b*** addresses this.

#3.1b In addition, the lysosomal SnxA signal appears to decrease with somewhat slower kinetics compared to PI3P. A short clarification as to whether MTM1 is expected to directly dephosphorylate PI(3,5)P_2, or whether the observed reduction in PI(3,5)P_2 is secondary to depletion of its precursor PI3P, resulting in gradual turnover of the PI(3,5)P_2 pool. Clarification of this point would further refine the mechanistic interpretation, although this point does not detract from the overall conclusions.

This specific point is now clearly discussed in the first results paragraph on **p. 2**: *"Note that initial reports of MTM1 activity reported selectivity for PI3P, with no activity against PI(3,5)P_2* (Taylor et al., 2000)*. Therefore, we cannot conclude whether PI(3,5)P_2 depletion occurs due to direct, MTM1-mediated degradation to PI5P (perhaps with slower kinetics than PI3P is converted to PI), or whether instead, the precursor PI3P is depleted, preventing synthesis of a rapidly turning-over pool of PI(3,5)P_2 (which again could therefore occur with slower kinetics than PI3P depletion)."*

#3.2. In Figure 3, the authors conclude that SnxA and FYVE do not overlap, but rather localize in close proximity. While this is an interesting observation, some degree of apparent overlap can be perceived in the images. Although the qualitative impression is largely convincing, inclusion of a

quantitative colocalization analysis (e.g., Pearson's or Manders' coefficients) could further strengthen this conclusion.

This is another important issue also raised by reviewer 1; it is specifically addressed in our response to ***#1.1c*** and ***#1.1d***.

#3.3. In Figure 4D, SnxA localization is still detectable in cells expressing the kinase-dead PIKfyve construct. If endogenous wild-type PIKfyve is present, residual PI(3,5)P_2 production could explain this signal and influence the interpretation of the experiment. The authors should clearly state which cell lines were used and whether endogenous PIKfyve activity was present in each condition. This information should be provided consistently in the figure legends to avoid ambiguity.

We have added the following clarification to the results section of ***"Design of a recruitable PIKfyve construct"*** **on p 3**: *"Note that for these experiments, the recruitable constructs are transiently expressed in unedited HEK293A cells which retain their native PIKfyve activity. Therefore, any induced PI(3,5)P_2 synthesis is expected to occur in addition to the baseline levels found in unperturbed cells."*

RE: Manuscript #E26-03-0120R
TITLE: "Quantitative comparison of PI(3,5)P2 biosensors reveals SnxA is the most sensitive and unbiased"

Dear Dr. Hammond:

Thank you for your very thoughtful rebuttal to the reviewers' comments, inclusion of new data to better vet the biosensors, and the direct discussion of caveats in the manuscript where appropriate. I agree that the manuscript has been strengthened by the revisions and recommend publication of the revised version of the manuscript.

Congratulations and thank you for submitting your work to Molecular Biology of the Cell! I do not doubt that your study will facilitate future work exploring the biology of this critical phosphoinositide.

Sincerely,
Laura Lackner
Monitoring Editor
Molecular Biology of the Cell

Dear Dr. Hammond:

Congratulations on the acceptance of your manuscript! Thank you for publishing your work in Molecular Biology of the Cell (MBoC).

Within 10 days, an unedited PDF of your manuscript will be published on MBoC in Press, an early-release journal version. The date your manuscript appears on this site is the official publication date.

Your copyedited and typeset manuscript will be scheduled for publication in the next available issue of MBoC. Once your paper is ready for review, our production team will notify you. In the summer of 2024, our production provider will introduce an online proofing solution, which will streamline the process of checking and validating author changes. This will result in improved typesetting quality, enhanced digital content, and a faster overall process.

We invite you to submit images related to your accepted manuscript to be considered for the journal cover.
Cover images must contain color, TIF Format
Size image at 7.4" wide x 5.85" deep
RGB and CMYK color (please submit two separate files, one in each format)
300 dpi resolution

Please also send a 1-paragraph description of the image that a general audience could understand. Include with the caption the name and institution of the individual(s) the image should be attributed to. Please provide this description in a Word file. You can send the file via email or by your favorite file transfer service to mboc@ascb.org.

Authors of Articles and Brief Communications are also encouraged to create a short video abstract to accompany their article when it is published. These video abstracts, known as Science Sketches, are up to 2 minutes long and will be published on YouTube and then embedded in the article abstract. Science Sketch Editors on the MBoC Editorial Board will guide you as you prepare your video. Information about how to prepare and submit a video abstract is available at www.molbiolcell.org/science-sketches. Please contact mboc@ascb.org if you are interested in creating a Science Sketch.

We look forward to publishing your paper in MBoC.

Sincerely,

Eric Baker
Journal Production Manager
MBoC Editorial Office
mbc@ascb.org
